# Fiberoptic bronchoscopy combined with urokinase therapy for the treatment of fatal massive hemoptysis: A case report

**DOI:** 10.1097/MD.0000000000036131

**Published:** 2023-11-17

**Authors:** Ju Huang, Qigang Zeng, Chengong Wei, Yong Dai

**Affiliations:** a The Department of Respiratory and Critical Care Medicine, Affiliated Guangdong Hospital of Integrated Traditional Chinese and Western Medicine, Foshan, Guangdong, China.

**Keywords:** fatal massive hemoptysis, fiberoptic bronchoscopy, urokinase

## Abstract

**Background:**

Fatal massive hemoptysis is a life-threatening emergency in the respiratory system. Currently, the treatment methods and techniques for massive hemoptysis are still limited, and there are often issues of delayed treatment or improper methods in clinical practice, leading to the difficulty of rescuing patients and high mortality rates. When fatal massive hemoptysis occurs, the key to successful treatment lies in whether intrapulmonary blood clots can be effectively cleared and airway patency can be ensured. Our practice of combining fiberoptic bronchoscopy with urokinase treatment to clear intrapulmonary blood clots after fatal massive hemoptysis demonstrates the effectiveness of this method.

**Case summary:**

We report a 32-year-old female who experienced cough, accompanied by fatal massive hemoptysis with extensive blood clot obstruction in the airway. Considering the difficulty of clearing the airway using conventional methods, it was decided to perform fiberoptic bronchoscopy combined with urokinase therapy after reviewing relevant literature. After treatment, the intrapulmonary blood clots were successfully extracted, thereby relieving airway obstruction. Finally, the patient was successfully weaned off extracorporeal membrane oxygenation, extubated, and evacuated from the ventilator. Currently, the patient’s condition is stable, and follow-up chest X-ray as well as computed tomography scans have shown improvement compared to previous assessments.

**Conclusion:**

Fatal massive hemoptysis is a intractable emergency in clinical practice. In this case, we confirmed that fiberoptic bronchoscopy combined with urokinase therapy may be effective and safe in the treatment of fatal massive hemoptysis.

## 1. Introduction

Fatal massive hemoptysis is a life-threatening emergency in the respiratory system, defined as any amount of hemoptysis that can cause airway obstruction, asphyxiation, and life-threatening situations, with the difficulty of accurately measuring the volume of blood loss during hemoptysis.^[1]^ Currently, the treatment methods and techniques for massive hemoptysis are still limited, and there are often issues of delayed treatment or improper methods in clinical practice, leading to the difficulty of rescuing patients and high mortality rates. When fatal massive hemoptysis occurs, the key to successful treatment lies in whether intrapulmonary blood clots can be effectively cleared and airway patency can be ensured. Here, we report a case of fatal massive hemoptysis admitted to the Intensive Care Unit of Guangdong Provincial Hospital of Integrated Traditional Chinese and Western Medicine.

## 2. Case presentation

**Figure 1.**
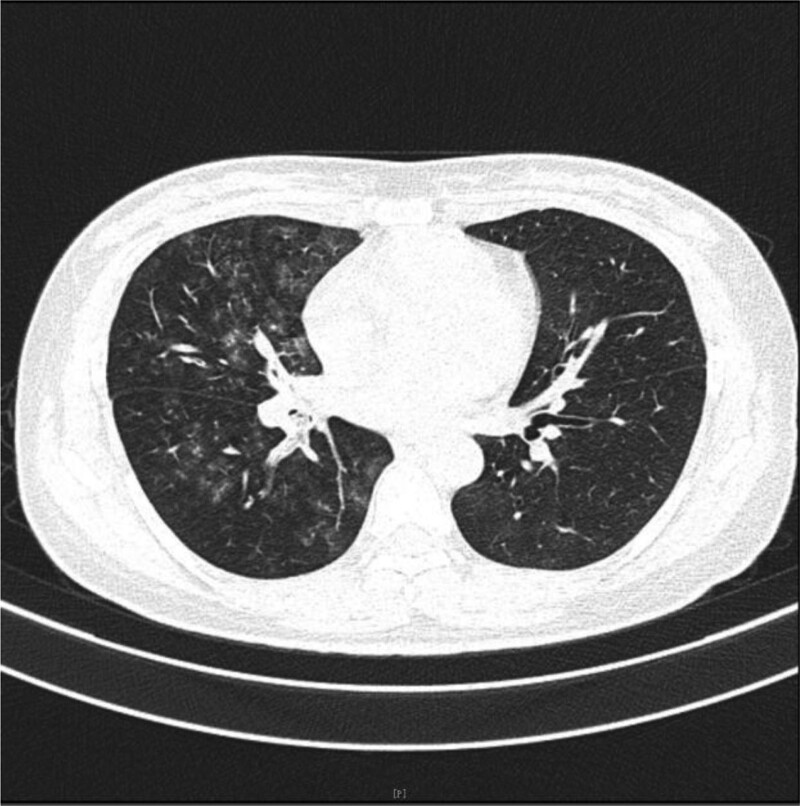
On May 17th, emergency chest CT showed multiple patchy and nodular ground-glass opacities in both lungs, with partial consolidation. The right lung was predominantly affected, showing some changes resembling the “paving stone” sign. Increased density opacities were observed within the right main bronchus, as well as the middle and lower lobes of the right lung. Bilateral pneumonia was considered, accompanied by alveolar hemorrhage, and multiple bronchial mucus plugs in the right main bronchus and bronchi of the middle and lower lobes.

**Figure 2.**
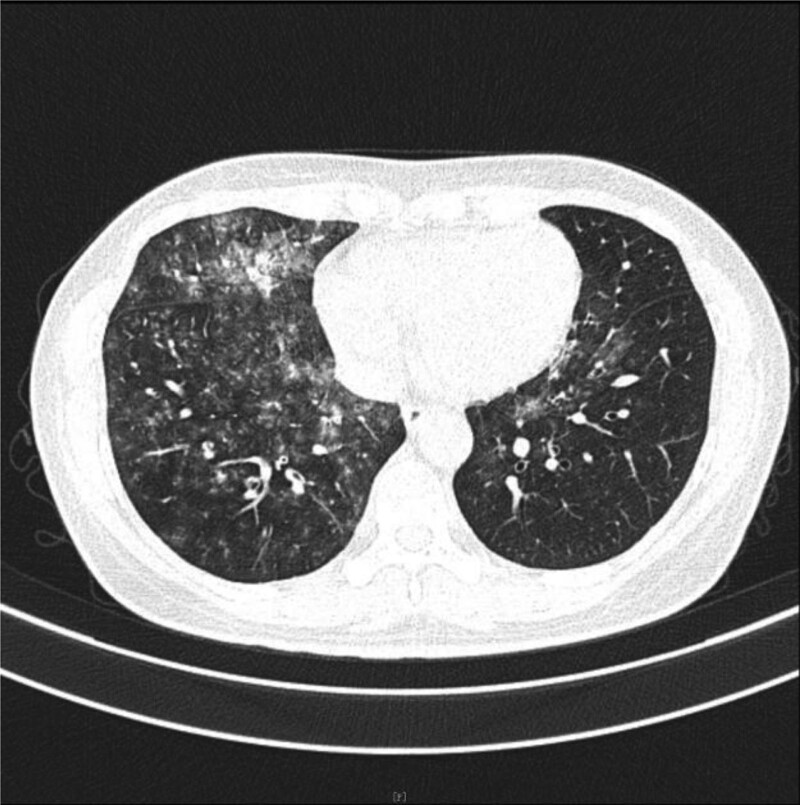
On May 17th, emergency chest CT showed multiple patchy and nodular ground-glass opacities in both lungs, with partial consolidation. The right lung was predominantly affected, showing some changes resembling the “paving stone” sign. Increased density opacities were observed within the right main bronchus, as well as the middle and lower lobes of the right lung. Bilateral pneumonia was considered, accompanied by alveolar hemorrhage, and multiple bronchial mucus plugs in the right main bronchus and bronchi of the middle and lower lobes.

The patient, a 32-year-old female, was admitted to the hospital on May 17th, 2023 at 15:51 with a chief complaint of “cough for 7 days, aggravated with hemoptysis for 9 hours.” She had a past medical history of Hashimoto thyroiditis, which was not systematically treated. She had had a cough for 7 days, which began to worsen 9 hours prior to admission, accompanied by 100 milliliters of blood. Chest computed tomography (CT) showed: (1) bilateral pneumonia with alveolar hemorrhage was considered, and reexamination was recommended after treatment. (2) Multiple bronchial mucus sputum plugs were found in the right main bronchus and the middle and lower lobes of the right lung (Figs. 1 and 2). In the afternoon of the same day, the patient developed hemoptysis again, with a large amount of fresh blood and a little blood clot. Physical examination: temperature: 36.2 °C, pulse: 128 beats/min, respiration: 25 breaths/min, blood pressure: 75/49 mm Hg, fingertip oxygen saturation: 50%. The breath sounds in the right lung were weak, while obvious moist rales were heard in the left lung.

## 3. Treatment

**Figure 3.**
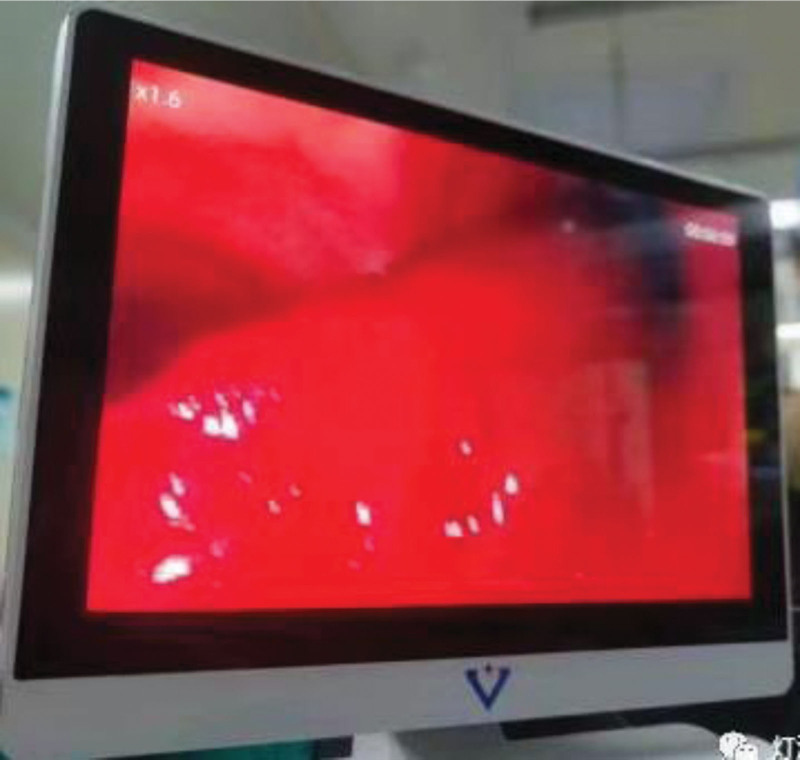
On May 17th, bedside fiberoptic bronchoscopy revealed a large amount of fresh blood within the airways, with extensive blood clots and blockage in both the left and right main bronchi.

Veno-venous extracorporeal membrane oxygenation (VVECMO) support was initiated along with assisted ventilation, blood transfusion, anti-infection treatment, mucolytics, bronchodilators, gastric acid suppression, and correction of electrolyte imbalances, providing comprehensive supportive care. Bedside fiberoptic bronchoscopy was applied. A large amount of blood was aspirated, and the left and right main bronchi were found to be blocked by clots (Fig. 3).

**Figure 4.**
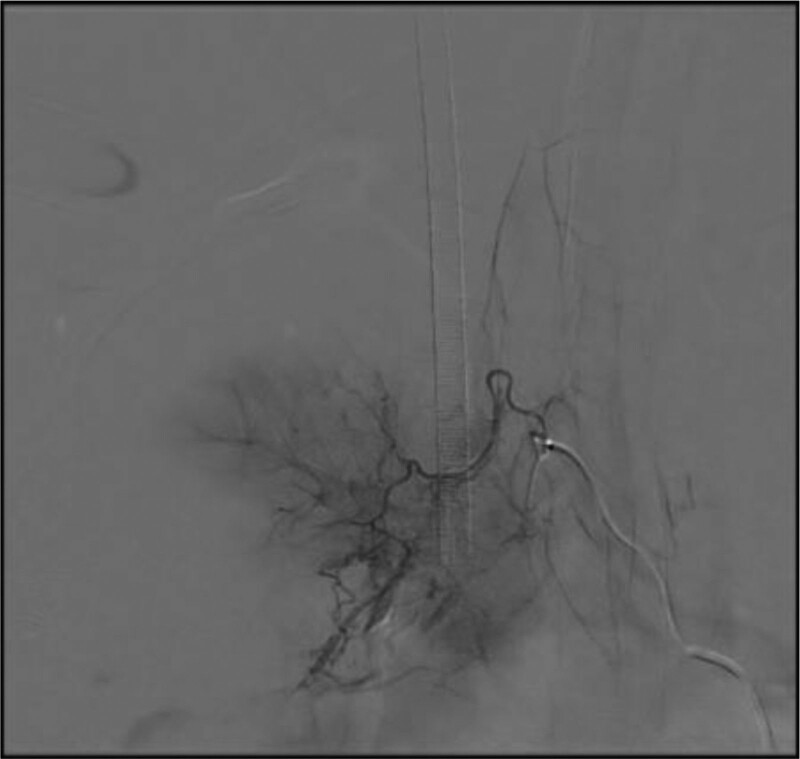
On the morning of May 18th, percutaneous bronchial artery angiography and embolization were performed. The angiography of the right pulmonary bronchial artery showed early venous manifestation, suggesting the presence of arteriovenous fistula. There were also signs of contrast agent staining, indicating the responsible vessel for the bleeding. Arterial embolization therapy was performed.

**Figure 5.**
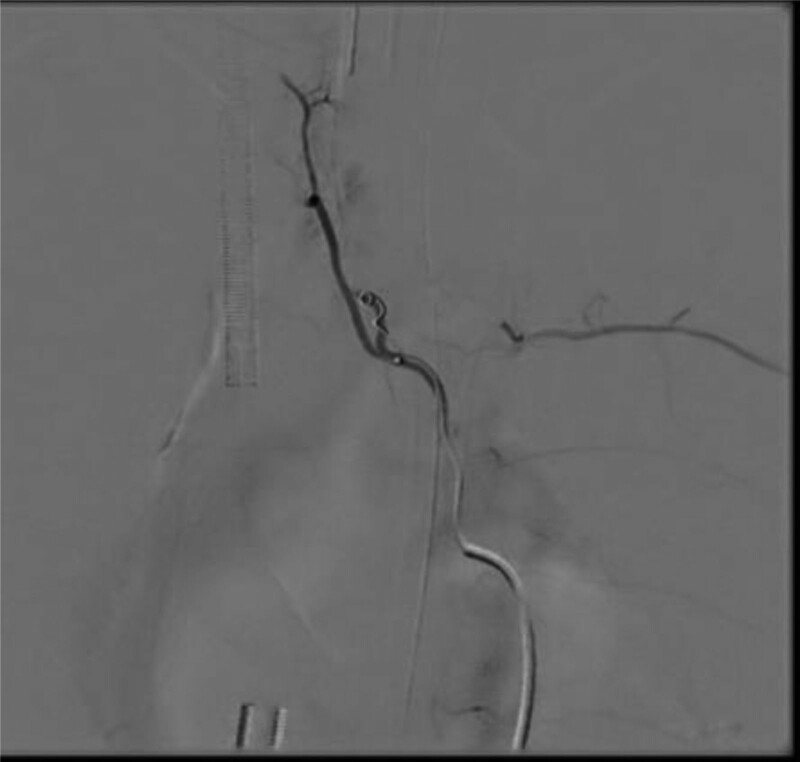
On the morning of May 18th, percutaneous bronchial artery angiography and embolization were performed. The angiography of the right pulmonary bronchial artery showed early venous manifestation, suggesting the presence of arteriovenous fistula. There were also signs of contrast agent staining, indicating the responsible vessel for the bleeding. Arterial embolization therapy was performed.

**Figure 6.**
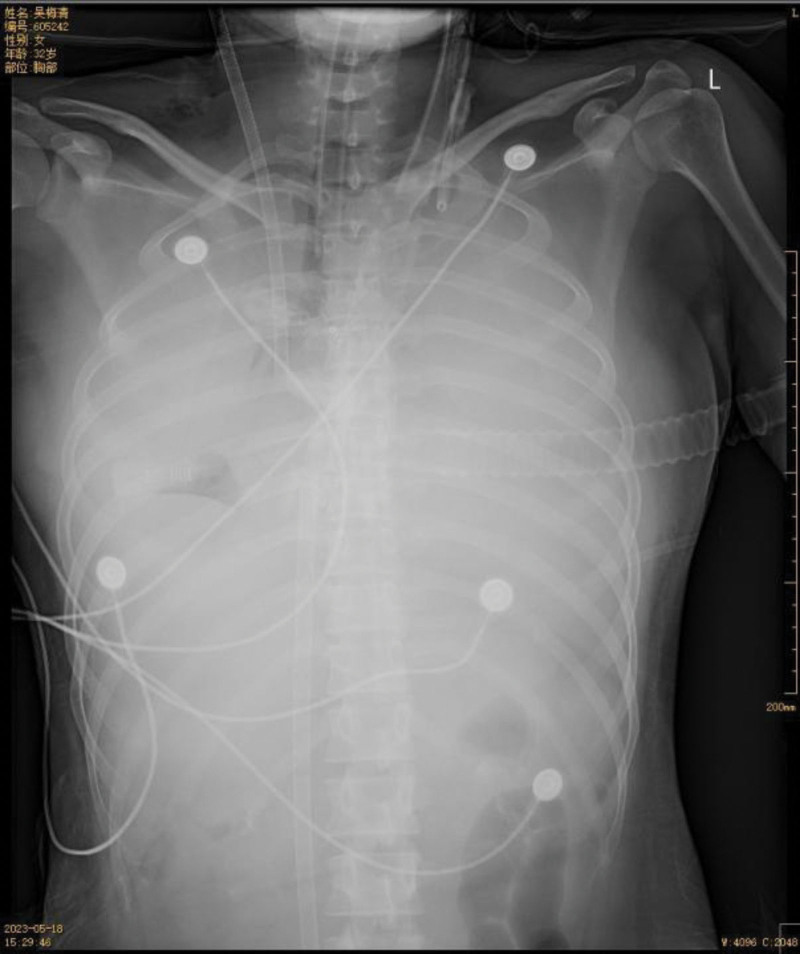
On the afternoon of May 18th, chest X-ray showed decreased volume and increased density in the right lung, while the left lung exhibited a large patchy high-density shadow resembling “white lung” appearance.

On May 18, the patient underwent percutaneous puncture bronchial artery angiography and embolization under VVECMO support and mechanical ventilation assistance. Pulmonary artery angiography revealed a faintly visible peripheral vascular network in the right middle and lower lungs, followed by arterial embolization therapy (Figs. 4 and 5). After the procedure, the tracheal bleeding was basically controlled, but there were significant amounts of residual blood clots and fibrinoid exudates in the bronchi of both lungs. The chest X-ray indicated “white lung”-like changes in both lungs (Fig. 6). The obstruction of the airway made it difficult to restore respiratory function, and it was challenging to achieve the desired tidal volume with mechanical ventilation assistance. Continuous VVECMO support was required to maintain oxygenation. The effective removal of intratracheal blood clots was crucial for the successful weaning and treatment of the patient. Therefore, bedside fiberoptic bronchoscopy was performed actively to clear the intrapulmonary blood accumulation. Despite repeated bronchoscopic lavage, negative pressure suction, forceps extraction, cryotherapy, as well as the combined use of supported laryngoscope, rigid bronchoscope, and bronchial endoscope, the clearance effect was unsatisfactory, and a large amount of blood clots in the airway was difficult to remove.

**Figure 7.**
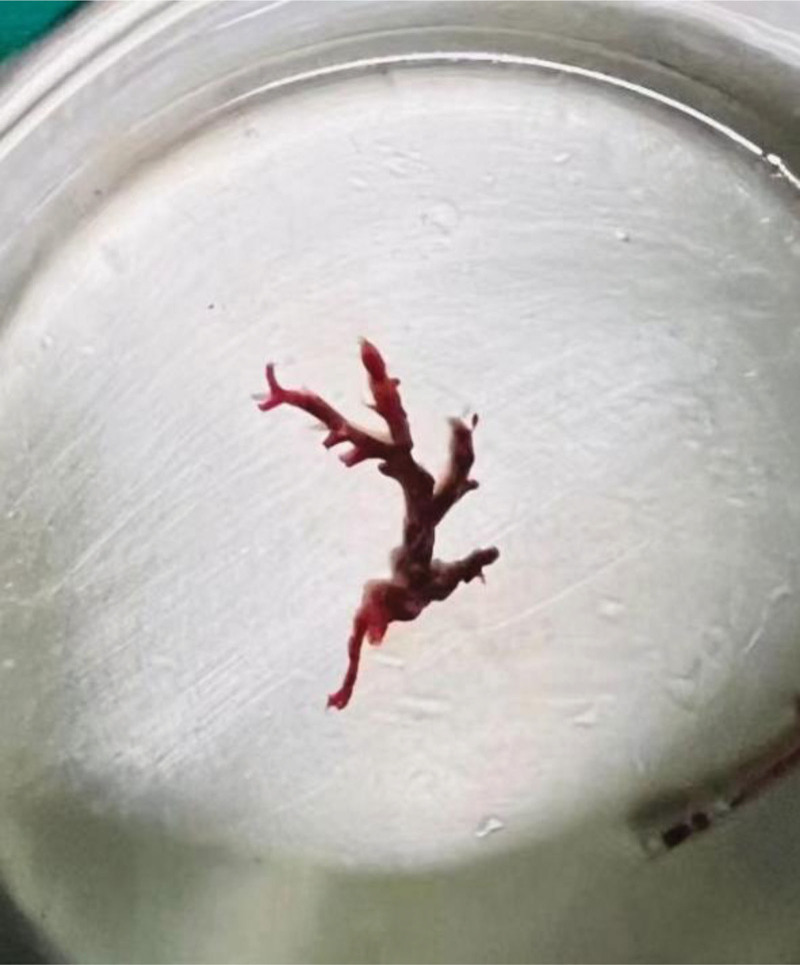
On the afternoon of May 18th, urokinase solution was instilled into the airways under fiberoptic bronchoscopy to induce clot dissolution. Subsequently, a large blood clot was successfully extracted using forceps, which showed bronchial tree like changes.

**Figure 8.**
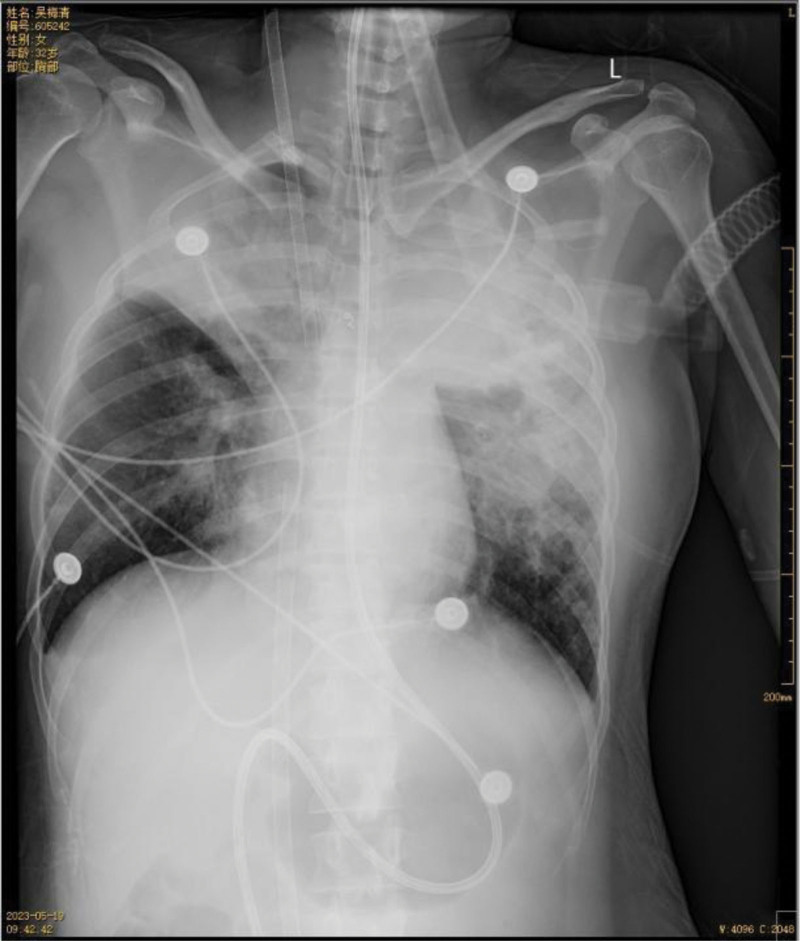
On May 19th, follow-up chest X-ray showed improvement in bilateral obstructive atelectasis compared to previous images.

After reviewing relevant literature, Song Fujie et al^[2]^ applied fiberoptic bronchoscopy combined with urokinase therapy to treat traumatic hemothorax. They dissolved 100,000 units of urokinase in 100 mL of normal saline and injected it into the adhered and difficult-to-remove blood clot in the thoracic cavity, and then used a fiberoptic bronchoscope to extract the clot. Among 17 patients, 15 were successfully cured without complications, resulting in a cure rate of 88.2%. This method has been proven to be effective and safe. Considering the difficulty of clearing the airway using conventional methods, a multidisciplinary consultation was conducted to fully evaluate the subsequent treatment options and their associated risks. It was decided to perform fiberoptic bronchoscopy combined with urokinase therapy. On May 18, 200,000 units of urokinase were administered via bronchoscopy, along with 500 mL of 0.9% saline solution for airway lavage, to promote clot dissolution. Subsequently, larger old blood clots and thrombus trees were successfully extracted using forceps (Fig. 7), thereby relieving partial airway obstruction. During the procedure, the patient’s vital signs and airway bleeding were closely monitored. The patient’s SPO2 was maintained at 100%, and hemodynamics remained stable. On May 19, a follow-up chest X-ray showed that the bilateral obstructive atelectasis was improved compared to before (Fig. 8).

Subsequently, bedside fiberoptic bronchoscopy was intermittently performed under extracorporeal membrane oxygenation support to clear the blood accumulation in the lung. Through repeated bedside fiberoptic bronchoscopy examinations and treatments, old blood clots and fibrinoid exudates were successively removed from the airway, resulting in a significant reduction in residual blood clots compared to before.

## 4. Outcome

**Figure 9.**
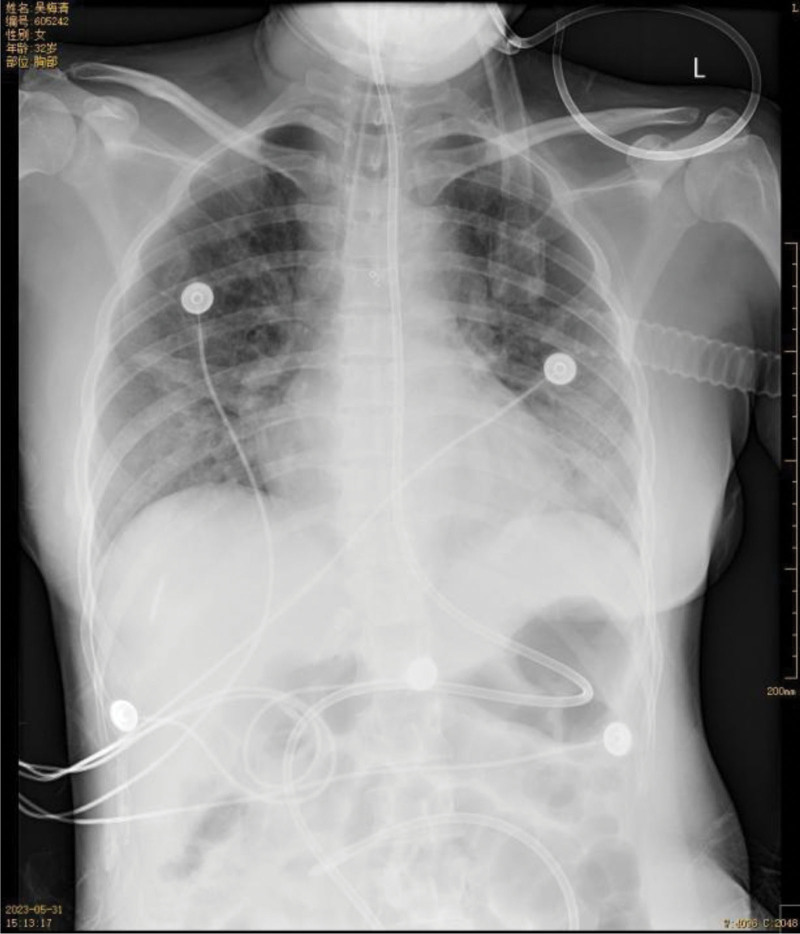
On May 31st, follow-up chest X-ray showed further improvement in bilateral obstructive atelectasis. The bilateral pneumonia and pulmonary hemorrhage had absorbed and reduced compared to previous images.

Following aggressive comprehensive treatment, the patient was successfully weaned off extracorporeal membrane oxygenation and evacuated from the ventilator. Currently, the patient’s condition is stable, and follow-up chest X-ray (Fig. 9) as well as CT scans have shown improvement compared to previous assessments.

## 5. Discussion

Massive hemoptysis is a common and critical condition in clinical practice. It can be caused by respiratory system diseases as well as multisystem diseases, such as infections, tumors, trauma, autoimmune diseases, hematologic diseases, etc. Among the various causes of hemoptysis, bronchiectasis, tuberculosis, pulmonary aspergillosis, necrotizing pneumonia, idiopathic hemoptysis, and lung cancer are the most common underlying factors.^[3,4]^ In this case, bronchial artery malformation was considered as the cause of hemoptysis, as the rupture of an artery due to severe coughing resulted in massive bleeding, which is uncommon in clinical practice.

A detailed collection of the patient’s medical history and comprehensive physical examination can provide clues for the etiological diagnosis of hemoptysis. Appropriate laboratory tests should be conducted based on the suspected causes to facilitate accurate diagnosis. Imaging examinations play a crucial role in identifying the underlying cause of hemoptysis. Chest X-ray is an important initial assessment tool,^[5]^ while contrast-enhanced chest CT scan can reveal pulmonary embolism, arteriovenous malformations, and arterial aneurysms. However, CT scans require a certain amount of time and the patient needs to maintain a supine position, which may increase the risk of asphyxiation, limiting its clinical utility.

Bronchoscopy is an essential tool for the diagnosis and treatment of hemoptysis.^[6,7]^ When the cause of hemoptysis is unknown, bronchoscopy should be performed as early as possible. In this case, the patient experienced massive hemoptysis in the emergency department, and bedside fiberoptic bronchoscopy was performed to assess the condition of the airway and determine the extent and location of the blood clot obstruction. Flexible bronchoscopy is relatively easy to manipulate, but its suction capacity is limited. On the other hand, rigid bronchoscopy can maintain airway patency and facilitate rapid suction,^[8]^ but it can only reach the main bronchi and requires general anesthesia, making the procedure more complex. Therefore, the selection of bronchoscopy technique should be based on the specific clinical scenario and available resources.

The main cause of death in massive hemoptysis is airway obstruction leading to asphyxia. Isolating the source of bleeding and ensuring airway patency are the key principles of hemoptysis management.^[9]^ When the bleeding is unilateral, the patient should be positioned on the affected side to prevent blood from flowing into the healthy lung and forming clots that blocks the airway. Medications used for hemostasis include pituitrin, hemocoagulase, adrenochromone tablets, ethamsylate, aminomethylbenzoic acid, vitamin K, and other hemostatic drugs. If the hemostatic effect of drugs is poor, bronchoscopy can be combined with other therapeutic interventions, such as endotracheal intubation on the healthy side, balloon tamponade in the bleeding airway, local epinephrine spraying, electrocautery, or cryotherapy for hemostasis. Bronchial artery embolization is also an effective hemostasis method with less trauma and has been widely used in clinical practice. When pharmacological and endoscopic treatments fail, bronchial artery embolization should be considered as a priority.^[10–12]^ In this case, the patient underwent bronchial artery embolization, which confirmed the injured artery and bleeding site based on bronchial arteriography and achieved immediate hemostatic effect. If bleeding cannot be controlled by multiple treatment and the bleeding is limited to one side of the lungs, surgical intervention may be considered. Of course, addressing the underlying cause is also an important aspect of managing hemoptysis.

The formation of massive blood clots that obstruct the airway is a challenging complication of hemoptysis and is one of the main factors contributing to poor prognosis. The key to successful treatment lies in the removal of blood clots, ensuring airway patency, and improving ventilation. Bronchoscopy is an important tool for clearing blood clots from the airway, and bedside fiberoptic bronchoscopy has been proven effective and easy to perform for airway management in critically ill patients. When conditions allow, a combination of flexible and rigid bronchoscopes can be used, and techniques such as negative pressure suction, balloon tamponade, biopsy forceps, and cryotherapy can be employed to maintain airway patency. In this case, the patient had massive blood clots and fibrinoid exudate obstructing the bronchi. Despite the use of conventional methods, such as negative pressure suction, forceps extraction, cryotherapy, and the combination of flexible and rigid bronchoscopes, clearing the airway of the massive blood clots was challenging. If the airway is not cleared promptly, the risk of death will increase. After reviewing relevant literature and drawing from the experience of Song Fujie et al,^[2]^ we decided to proceed with bronchoscopy-guided urokinase irrigation based on a thorough evaluation of the subsequent treatment options and risks. Urokinase is widely used in clinical practice for thrombolytic therapy in thrombotic diseases. It catalyzes the conversion of plasminogen into plasmin, which degrades fibrin clots. Moreover, urokinase appears to be a more effective fibrinolytic agent than other agents and have a lower rate of adverse events.^[13,14]^ In this case, urokinase irrigation was performed under bronchoscopy, leading to the degradation of the blood clots and making them easier to remove, ultimately relieving airway obstruction and achieving a definite therapeutic effect.

Prior to treatment, it is important to utilize relevant imaging examinations to identify the specific irrigation site. Attention should be paid to the risk of bleeding, and necessary preparations should be made. During the treatment, close monitoring of the patient’s vital signs and changes in their condition is crucial. If necessary, the treatment should be immediately terminated. Care should be taken to avoid rough movements during the procedure, and difficult-to-remove blood clots should not be forcibly grasped, so as to avoid bleeding. Under direct visualization with a fiberoptic bronchoscope, a portion of urokinase solution can be directly injected into the clot to facilitate its dissolution before attempting removal. Additionally, proactive and efficient multidisciplinary collaboration is also the key to gaining time and opportunities for subsequent treatment.

Rarely reported in the literature, the treatment method of urokinase irrigation under bronchoscopy is explored in this case. The practice of combining fiberoptic bronchoscopy with urokinase treatment to unblock the airway obstructed by fatal massive hemoptysis demonstrates the effectiveness of this method, which is both feasible and easy to perform. In clinical practice, fatal massive hemoptysis often results in the formation of blood clots that block the airway. Sometimes the conventional treatment methods may fail to achieve satisfactory results. In such cases, after a thorough risk assessment, exploratory treatment can be considered to win the critical time window of survival and create conditions for the subsequent treatment to improve the prognosis of patients.

However, this study has some limitations. One limitation is the short follow-up time. Although the patient have a good quality of life now, further patient follow-up and regular chest radiography or chest CT is necessary. Another limitation is the limited number of cases. As a result, this treatment remains to be further observed and validated.

## 6. Conclusion

Fatal massive hemoptysis is characterized by its urgent onset, rapid progression, critical condition, and the difficulty in emergency rescue. It requires clinicians to make accurate judgments quickly and initiate effective treatment as soon as possible. This case confirms that fiberoptic bronchoscopy combined with urokinase therapy is effective and safe for removing intrapulmonary blood clots after fatal massive hemoptysis. The successful application is expected to provide reference experience and ideas for the treatment of such cases in clinical practice.

## Author contributions

**Conceptualization:** Ju Huang, Qigang Zeng, Chengong Wei, Yong Dai.

**Data curation:** Ju Huang, Qigang Zeng.

**Formal analysis:** Ju Huang, Qigang Zeng.

**Funding acquisition:** Ju Huang, Qigang Zeng, Chengong Wei, Yong Dai.

**Investigation:** Ju Huang, Qigang Zeng.

**Methodology:** Ju Huang, Qigang Zeng.

**Project administration:** Ju Huang, Qigang Zeng, Chengong Wei, Yong Dai.

**Resources:** Ju Huang, Qigang Zeng, Chengong Wei, Yong Dai.

**Software:** Ju Huang, Qigang Zeng.

**Supervision:** Ju Huang, Qigang Zeng, Chengong Wei, Yong Dai.

**Validation:** Ju Huang, Qigang Zeng, Chengong Wei, Yong Dai.

**Visualization:** Ju Huang, Qigang Zeng.

**Writing – original draft:** Ju Huang, Qigang Zeng.

**Writing – review & editing:** Ju Huang, Qigang Zeng.
